# Musical Amnesia

**Published:** 1893-01-07

**Authors:** 


### MUSICAL AMNESIA.

Dr. Brazier in the *Revue Philosophique* discusses the disease of amnesia as distinct from aphasia. He shows it to exist in three forms, corresponding to the three methods in which the calling up of a musical image in the brain may be effected. In the case of an "auditive image" the melody is heard mentally. This faculty may be altogether wanting as in an instance cited from Mr. Grant Allen of a young man to whom all the notes of the piano sounded the same, though in other respects his hearing was not deficient. Or the faculty may be suddenly lost, as in the case of the eminent pianist Prudent, who while executing a concerto of his own with orchestral accompaniment, lost abruptly the memory of everything connected with music, so that his work was no more for him than an incoherent noise. Where the image is "visual," the musician will read the melody or see it written before him, mentally. A case of visual amnesia is cited where an accomplished pianiste found herself suddenly unable to play from notes or memory on account of the failure of the visual images on which she completely depended. The musical notation no longer conveyed any meaning to her. "Motive amnesia" again depends on a third method of calling up the musical image, viz., by playing or singing it mentally. An instance is cited from Dr. Charcot of a trombone player who, with his other motive powers and memories remaining intact, had lost the movements associated with the hand and the mouth necessary to the playing of his instrument. The young tenor who retained all his musical faculties yet could never sing true again after an accident to the brain is an example of "motive amnesia" affecting the organs of the voice. Dr. Brazier argues that since amnesia depends on a defect of action in the registrating centres and producers of images, these centres presiding over the different symbols would be localised in cortical cellular groups, very near to one another but nevertheless distinct, and he concludes that the facts hitherto ascertained fully justify the application of this theory of the three images to music and its mental representations.

